# Position effect, cryptic complexity, and direct gene disruption as disease mechanisms in *de novo* apparently balanced translocation cases

**DOI:** 10.1371/journal.pone.0205298

**Published:** 2018-10-05

**Authors:** Constantia Aristidou, Athina Theodosiou, Mads Bak, Mana M. Mehrjouy, Efthymia Constantinou, Angelos Alexandrou, Ioannis Papaevripidou, Violetta Christophidou-Anastasiadou, Nicos Skordis, Sophia Kitsiou-Tzeli, Niels Tommerup, Carolina Sismani

**Affiliations:** 1 Department of Cytogenetics and Genomics, The Cyprus Institute of Neurology and Genetics, Nicosia, Cyprus; 2 The Cyprus School of Molecular Medicine, The Cyprus Institute of Neurology and Genetics, Nicosia, Cyprus; 3 Wilhelm Johannsen Centre for Functional Genome Research, Department of Cellular and Molecular Medicine, University of Copenhagen, Copenhagen N., Denmark; 4 Department of Clinical Genetics, The Cyprus Institute of Neurology and Genetics and Archbishop Makarios III Medical Centre, Nicosia, Cyprus; 5 Division of Pediatric Endocrinology, Paedi Center for Specialized Pediatrics, Nicosia, Cyprus; 6 St George’s University of London Medical School at the University of Nicosia, Nicosia, Cyprus; 7 Department of Medical Genetics, Medical School, University of Athens, Athens, Greece; Virginia Commonwealth University, UNITED STATES

## Abstract

The majority of apparently balanced translocation (ABT) carriers are phenotypically normal. However, several mechanisms were proposed to underlie phenotypes in affected ABT cases. In the current study, whole-genome mate-pair sequencing (WG-MPS) followed by Sanger sequencing was applied to further characterize *de novo* ABTs in three affected individuals. WG-MPS precisely mapped all ABT breakpoints and revealed three possible underlying molecular mechanisms. Firstly, in a t(X;1) carrier with hearing loss, a highly skewed X-inactivation pattern was observed and the der(X) breakpoint mapped ~87kb upstream an X-linked deafness gene namely *POU3F4*, thus suggesting an underlying long-range position effect mechanism. Secondly, cryptic complexity and a chromothripsis rearrangement was identified in a t(6;7;8;12) carrier with intellectual disability. Two translocations and a heterozygous deletion disrupted *SOX5*; a dominant nervous system development gene previously reported in similar patients. Finally, a direct gene disruption mechanism was proposed in a t(4;9) carrier with dysmorphic facial features and speech delay. In this case, the der(9) breakpoint directly disrupted *NFIB*, a gene involved in lung maturation and development of the pons with important functions in main speech processes. To conclude, in contrast to familial ABT cases with identical rearrangements and discordant phenotypes, where translocations are considered coincidental, translocations seem to be associated with phenotype presentation in affected *de novo* ABT cases. In addition, this study highlights the importance of investigating both coding and non-coding regions to decipher the underlying pathogenic mechanisms in these patients, and supports the potential introduction of low coverage WG-MPS in the clinical investigation of *de novo* ABTs.

## Introduction

The great majority of apparently balanced translocation (ABT) cases are phenotypically normal since theoretically there is no obvious loss or gain of genetic material. However, association with specific clinical phenotypes has been originally estimated in 6–10% of *de novo* ABT cases [1], while recent studies assessing long-term outcomes in *de novo* ABT carriers estimated a morbidity risk of 27% [2].

Many molecular mechanisms have been suggested to account for clinical phenotypes observed in ABT carriers. First of all, this may be due to direct disruption of dosage-sensitive genes [3], which also exhibit haploinsufficiency [4]. Apart from direct protein-coding gene disruption, ABT breakpoints can also map in intragenic areas, and may affect the regulation of nearby genes by disrupting *cis*-regulatory elements, such as enhancers, through long-range position effect (LRPE) [5,6]. For example, ABTs may affect the regulatory landscape of genes by disrupting highly-conserved topologically associated domains (TADs) [7] within which physical contact of genes and their regulators is achieved, via chromatin looping, for correct expression to occur [8]. Specifically, a translocation can remove an enhancer/silencer from its target gene or place a gene next to an enhancer/silencer that is not its own, therefore leading to gene repression, overexpression, or ectopic expression. A third mechanism underlying phenotype presentation in ABT carriers is the presence of cryptic genomic imbalances near the translocation breakpoints or elsewhere in the genome [9,10], or the presence of complex chromosomal rearrangements (CCRs) [9–11]. Other rarer molecular mechanisms include disruption of an imprinting locus in uniparental disomy (UPD) cases [12], unmasking of recessive gene variants by loss-of-function at the translocation breakpoints leading to functional homozygosity [13], as well as gene fusion generation which may result in a novel, non-functional or pathogenic protein [14]. Finally, the presented phenotypes in ABT carriers may be unrelated to the translocation and simply coincidental [15].

Detailed characterization of translocations and accurate breakpoint mapping in affected ABTs carriers are crucial for positioning disease-candidate genes at or near the breakpoints [16]. The overall purpose of this study was the use of Whole-Genome Mate-Pair Sequencing (WG-MPS) (low coverage) to decipher the pathogenic molecular mechanisms underlying phenotype associations in three affected *de novo* ABT cases. Based on the results, a causative link between translocation rearrangements and phenotype presentation in affected *de novo* ABT carriers was established.

## Materials and methods

### Bioethics statement and consent form collection

This study was approved by the National bioethics committee as part of the Translation Facility Application with number EEBK/EII/2-13/09. Written informed consent was obtained from all patients prior to initiation of the study.

### Whole-genome mate-pair sequencing and translocation validation

In the current study, 1μg of high quality DNA sample from each ABT case was used to prepare WG-MPS libraries (~2-4kb insert length) by following the Nextera Mate-Pair Sample Preparation Guide, gel-free protocol (Part #15035209 Rev.C) from Illumina (http://www.illumina.com/). WG-MPS library preparation, library pooling, sequencing on an Illumina HiSeq2500 system, data analysis and filtering, translocation breakpoint validation, as well as LRPE investigation were performed as described previously [15]. PCR primers used for translocation breakpoint validation are available in S1 Table.

### Depth of coverage analysis

In order to identify any imbalances as a change in the depth of coverage of the WG-MPS paired-end reads, the WG-MPS aligned files from selected samples were used as an input in the cnv tool incorporated in the SVDetect software [17]. This tool calculates depth of coverage log ratios from an affected (patient) and a non-affected (reference) sample. The log ratio data were then used as input in order to create density plots in CIRCOS, a software package to visualize data in circular output [18].

### X-inactivation analysis

The X-chromosome inactivation status in a female patient (Case 1) carrying an X:autosome translocation was determined by methylation analysis of the human androgen receptor (*AR*) gene at Xq11.2, as described previously [19]. PCR products were run using a Fragment Analysis protocol on a 3130xl Genetic Analyzer (Applied Biosystems), and X-inactivation analysis results were analyzed by using the Microsatellite application of the GeneMapper v4.1 Software (Applied Biosystems), according to the manufacturer’s instructions.

### RNA extraction and DNase treatment

Total RNA from Epstein-Barr virus-transformed lymphoblastoid cell lines from patients and a control sample was extracted with RNeasy Midi Kit (Qiagen, Hilden, Germany) following the manufacturer’s instructions, and RNA concentration was measured with an ND-1000 Spectrophotometer (Thermo Fisher Scientific, Waltham, MA, USA). For reverse transcription PCR (RT-PCR) experiments, 10μg RNA was treated with DNase I (New England Biolabs (NEB), Ipswich, MA, USA) to remove residual genomic DNA according to the manufacturer’s protocol.

### Reverse-transcription PCR primer design and procedure

RT-PCR primer pairs (Metabion, Planegg, Germany) were designed using the Primer3 web interphase tool [20]. Wherever possible, forward and reverse primers were designed on two consecutive exons spanning an intermediate large intron of the gene of interest. If a gene of interest has only one protein-coding exon and no intermediate introns, RT-PCR primer pairs were designed within the same exon. DNase-treated RNA (10pg-1μg) was reverse transcribed using the ProtoScript First Strand cDNA Synthesis Kit (NEB) with oligo-dT primers, according to the kit’s manual. ~50ng cDNA from each sample was amplified by 40 cycles of PCR using pre-designed RT-PCR primer pairs within the genes of interest. An RNA sample not reverse transcribed and a no template sample were used as negative controls. Finally the integrity of the cDNA samples was shown by amplification of a 215bp fragment from the housekeeping gene *ACTB* (β-actin). RT-PCR primer pairs and PCR conditions are available on request.

## Results

By using WG-MPS, translocation breakpoint junctions in all three affected *de novo* ABT carriers included in the present study were successfully identified and mapped down to a region ranging between 407bp and 1.9kb (Table 1). Direct and indirect disease-candidate gene disruption occurred in 2/3 and 1/3 cases, respectively, while cryptic complexity was identified in 1/3 cases. Microhomology (1-5bp) and imbalances (1-6bp) were also detected at the translocation breakpoint sites (Table 1). Detailed results are presented below:

**Table 1:** Translocation breakpoints as identified by Whole-Genome Mate-Pair Sequencing and validated by Sanger sequencing (hg19).

Translocation junctions as estimated by WG-MPS	Junction Length	Translocation breakpoint position as defined by SS	Disrupted Genes	Insertions/deletions (+ strand)	Microhomology (+ strand)
Case 1—Female with hearing loss					
chr1:74499775–74500253	479bp	chr1:74500101–74500108	*LRRIQ3*	AATTCA duplication	AATTC
chrX:82675876–82676282	407bp	chrX:82676074–82676079	~87kb upstream *POU3F4*	GAATT duplication G deletion	GAATT
Case 2—Female with mild to moderate intellectual disability					
chr6:16754266–16755111	845bp	chr6:16754305–16754306	*ATXN1*	ND	-
chr7:120530428–120531641	1214bp	chr7:120530522–120530523	-	ND	G
chr7:124121849–124122971	1123bp	chr7:124122363–124122371	-	ATCTTTT deletion T insertion	-
chr8:129588349–129589617	1269bp	chr8:129589008–129589009	-	ND	CTGG
chr8:132602314–132603246	933bp	chr8:132602994–132602995	-	-	-
chr8:132899241–132900431	1191bp	chr8:132899713–132899714	-	T insertion	-
chr8:132937706–132938936	1231bp	chr8:132938698–132938699	*EFR3A*	ND	-
chr12:24016019–24017286	1268bp	chr12:24016112–24016113	*SOX5*	ND	C
chr12:27177142–27177824	682bp	chr12:27177486–27177487	*MED21*	ND	-
chr12:55357036–55357802	767bp	chr12:55357553–55357554	*TESPA1*	ND	CCAG
chr12:65386366–65387285	920bp	chr12:65386836–65386837	-	ND	-
chr12:68784020–68784572	553bp	chr12:68784362–68784363	-	ND	-
Case 3—Female with dysmorphic facial features and speech delay					
chr4:118195375–118197310	1936bp	chr4:118195456–118195460	-	AGG duplication	G
chr9:14104333–14104994	662bp	chr9:14104378–14104384	*NFIB*	GTCTA deletion	-

The junction length, disrupted genes, as well as insertions/deletions and microhomology at the breakpoint junctions are also given. In Case 2, only those translocation breakpoint junctions mapped to the base-pair level are included in Table 1. Due to the highly repetitive regions at most chromothripsis rearrangement breakpoints in Case 2, validation was not feasible for all breakpoint junctions, and hence, possible insertions/deletions could not be determined in most cases. (bp = base-pairs; dn = *de novo*; WG-MPS = Whole-Genome Mate-Pair Sequencing; ND = not determined; SS = Sanger sequencing)

### Case 1—46,X,t(X;1)(q13;p31)dn

**Fig 1:**
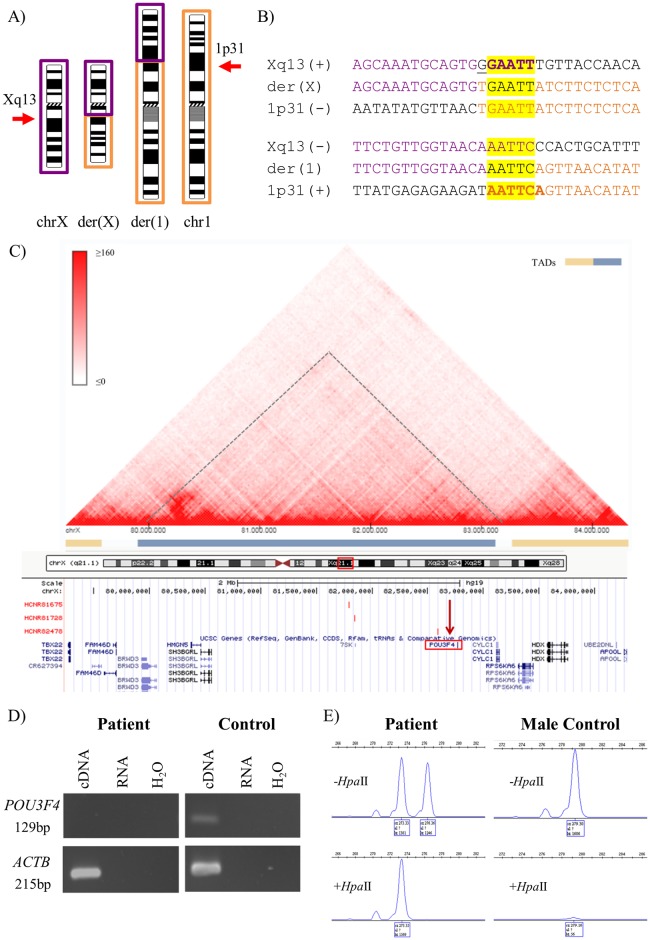
Summarized karyotype, WG-MPS, Sanger sequencing, RT-PCR and X-inactivation results from Case 1. A) Partial ideograms showing the normal and derivative (der) chromosomes (chr) X (purple) and 1 (orange) (not to scale) in Case 1. Xq13 and 1p31 breakpoints are indicated by arrows. B) Derivative translocation junction sequences (middle line) and matching reference sequences (top and bottom lines) as identified by Sanger sequencing. Microhomology is highlighted in yellow, deleted sequences are underlined, and duplicated sequences are in bold letters. C) Schematic illustration of the Topologically Associated Domain (TAD) structure at the *POU3F4* locus (indicated with a dashed line and a blue bar) as created by the 3D Genome Browser (http://promoter.bx.psu.edu/hi-c/). Each dot within individual TADs reflects the interaction between two DNA positions. The corresponding region from the UCSC Genome Browser was aligned underneath the heat map. This illustrates among other genes *POU3F4* (red rectangle), the der(X) breakpoint identified in the current study mapping ~87kb upstream *POU3F4* (vertical red arrow), as well as specific enhancers identified by Naranjo *et al*., 2010 upstream *POU3F4* (red vertical bars). D) RT-PCR results showing absence of *POU3F4* expression in the patient as compared with a normal control. cDNA = RNA reverse transcribed; RNA = RNA not reverse transcribed; H_2_O = no template sample. E) X-inactivation analysis results showing a skewed X-inactivation pattern (100:0). A PCR product is obtained only from the inactive (methylated) chromosomes. After *Hpa*II digestion, only one peak was shown in the female patient, whereas, no peak was observed in the male control.

Case 1 is a female patient with hearing loss and a *de novo* t(X;1)(q13;p31) translocation (Fig 1A) as detected by previous karyotype and FISH analyses. WG-MPS followed by Sanger sequencing accurately identified and mapped both translocation breakpoints (Fig 1B; Table 1). The der(1) breakpoint disrupted the non-pathogenic leucine rich repeats and IQ motif containing 3 (*LRRIQ3*) gene (intron 7/7) (NM_001105659.1), while the der(X) breakpoint mapped ~87kb upstream the POU class 3 homeobox 4 (*POU3F4*) gene (OMIM-300039) (NM_000307.4) (Fig 1C; Table 1). Mutation screening revealed no variants in the coding sequence of *POU3F4* apart from two benign single nucleotide polymorphisms (rs5921978 and rs5921979; S1 Fig). In addition, RT-PCR analysis revealed absence of *POU3F4* expression in the patient as compared with a normal control (Fig 1D). Finally, a highly skewed X-inactivation pattern (100:0) was observed in Case 1 after pre-digestion with the methylation-sensitive restriction enzyme *Hpa*II (Fig 1E).

### Case 2—46,XX,t(6;7;8;12)(p22.2;q31.3;q24.3;q13.1)dn

**Fig 2:**
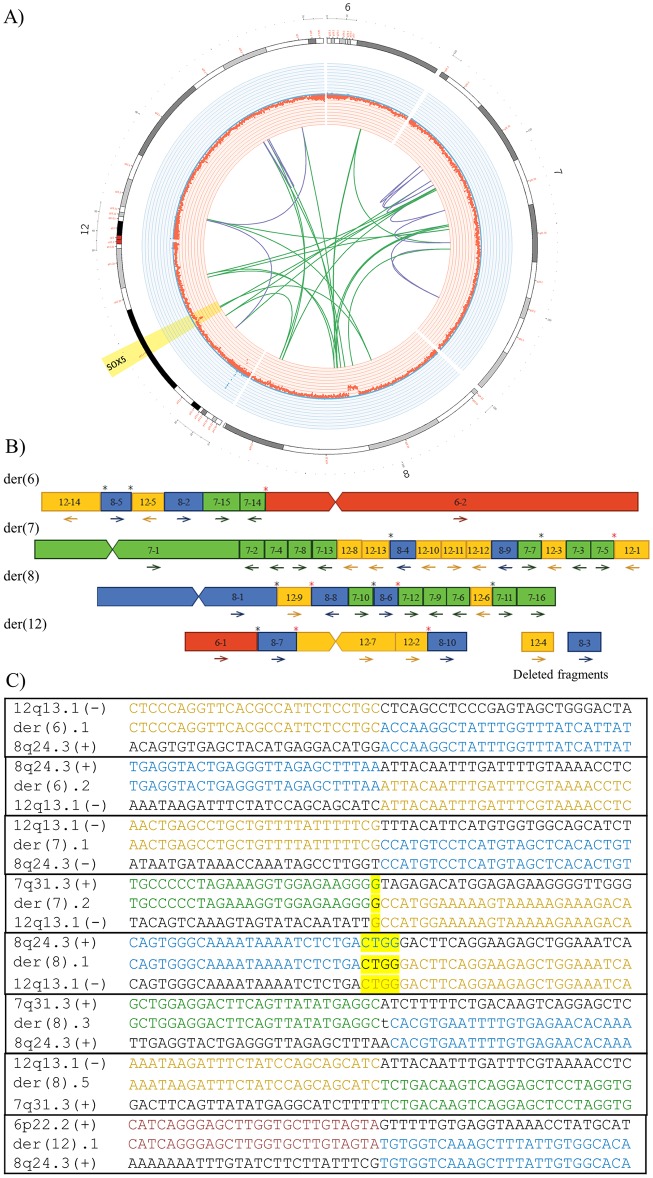
Summarized WG-MPS and Sanger sequencing results in Case 2. A) Circos plot illustrating partial chr6, chr7, chr8, and chr12 ideograms (outer circle), deletions (red) and duplications (blue) based on the depth of coverage analysis (inner circle) as well as inter-translocation (green lines) and intra-translocation (purple lines) junctions identified by WG-MPS in Case 2. *SOX5* disruption by a heterozygous deletion and two inter-translocation breakpoints is highlighted. B) Derivative chromosome reconstruction of the chromothripsis rearrangement. Fragments from chromosomes 6, 7, 8, and 12 are in red, green, blue, and yellow, respectively. Arrows: fragment orientations; black asterisks: translocation junctions sequenced to the base-pair level; red asterisks: translocation junctions amplified but not sequenced. C) Derivative translocation junction sequences (middle line) and matching reference sequences (top and bottom lines) as identified by Sanger sequencing in Case 2. Microhomology is highlighted, and inserted sequences not aligning to either chromosome are in lower-case letters.

Case 2 is an affected female presenting with mild to moderate intellectual disability (ID). Initial G-banding and FISH analyses detected a *de novo* apparently balanced CCR involving chromosomes 6, 7, 8, and 12, and seven breakpoints [11]. Previous array-CGH revealed a heterozygous deletion on chromosome 8q24.22; arr[GRCh37] 8q24.22(131465786_132161678)x1, overlapping the adenylate cyclase 8 (*ADCY8*) (OMIM-103070) gene. This chr8 deletion was also identified from the depth of coverage analysis in this study, which additionally detected a patient-specific ~108kb heterozygous deletion on 12p12.1 overlapping exons 1–3 of the SRY-box 5 (*SOX5*) gene (OMIM-604975) (NM_006940.4) (Fig 2A). Interestingly, by using WG-MPS, cryptic complexity and a chromothripsis rearrangement was revealed including thirty-eight interchromosomal and intrachromosomal translocation junctions in total (Fig 2A) (Table 1; S2 Table). After accurate derivative chromosome reconstruction of the complex rearrangement (Fig 2B), a number of translocation breakpoints (marked with a black asterisk in Fig 2B) were validated by Sanger sequencing (Fig 2C; Table 1). In the remaining translocation breakpoint junctions, sequence-specific PCR primer design was challenging as most were spanning very long repeats. In some of these cases, a PCR product was obtained by long-range PCR; however, breakpoint mapping to the base-pair level with Sanger sequencing was not feasible (marked with a red asterisk in Fig 2B).

### Case 3—46,XX,t(4;9)(q26;p24)dn

**Fig 3:**
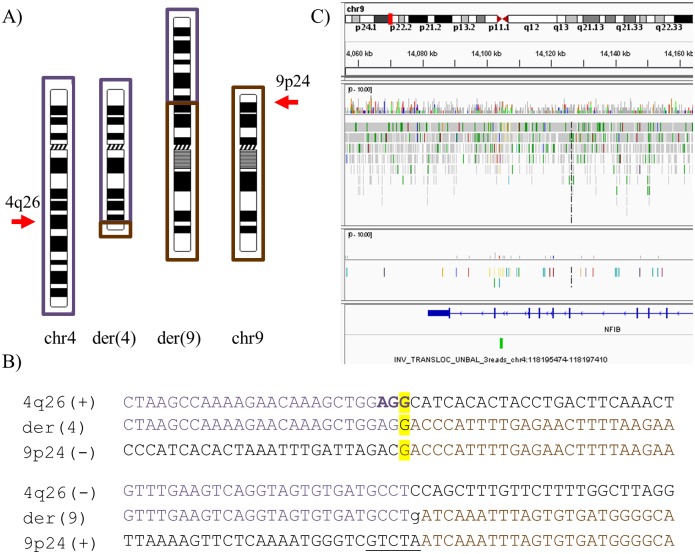
Summarized WG-MPS and Sanger sequencing results in Case 3. A) Partial ideograms showing the normal and derivative (der) chromosomes (chr) 4 (dark purple) and 9 (brown) (not to scale) in Case 3. 4q26 and 9p24 breakpoints are indicated by arrows. B) Derivative translocation junction sequences (middle line) and matching reference sequences (top and bottom lines) as identified by Sanger sequencing. Microhomology is highlighted in yellow, deleted sequences are underlined, duplicated sequences are in bold letters, and inserted sequences not aligning to either chromosome are in lower-case letters. C) IGV screenshot illustrating the der(9) breakpoint disrupting the *NFIB* gene within intron 10/10 (NM_001190737.1).

Case 3 is a female patient presenting with dysmorphic facial features and speech delay. Initial karyotype analysis detected a *de novo* apparently balanced t(4;9)(q26;p24) translocation (Fig 3A). FISH analysis confirmed the rearrangement, while array-CGH at 1MB resolution revealed no chromosomal imbalances. In the present study, WG-MPS followed by Sanger sequencing accurately detected the t(4;9) translocation and mapped both breakpoints (Fig 3B; Table 1). The der(4) breakpoint did not disrupt any genes, whereas the der(9) breakpoint directly disrupted intron 10/10 of the Nuclear Factor I B (*NFIB*) gene (OMIM-600728) (NM_001190737.1) (Fig 3C).

## Discussion

In contrast to our recent findings concerning familial cases with identical ABTs and discordant phenotypes [15], breakpoint mapping results from the present study demonstrated that translocations appear to be correlated with phenotype presentation in *de novo* ABT cases. This is achieved through a number of underlying molecular mechanisms including LRPE, cryptic complexity, and direct disease-associated gene disruption by the translocation breakpoints.

In the ABT Case 1 with hearing loss and a t(X;1) translocation, WG-MPS mapped the der(X) breakpoint ~87kb upstream the *POU3F4* gene (also known as *Brain-4* or *BRN-4*) (NM_000307.4) (Fig 1C; Table 1). This gene encodes for the POU domain, class 3, transcription factor 4, which is important for the development of the inner ear [21]. Missense, nonsense, and frameshift variants as well as deletions overlapping the *POU3F4* gene have been identified in patients with X-linked recessive deafness [22–24]. Structural variants upstream the coding *POU3F4* region, including microdeletions, inversions, and duplications, have also been reported in patients with similar hearing loss phenotypes, thus indicating the presence of *cis*-regulatory elements (e.g. enhancers) within those regions (even up to 900kb upstream), which if disturbed result in altered *POU3F4* expression through LRPE [25–27]. The majority of *POU3F4* disruption cases have been reported in affected males. In contrast, female carriers are rarely reported, and in these cases, normal, late-onset, or progressive mild to moderate hearing loss is usually observed [28]. This is due to the presence of a second, normal X-chromosome in females and random X-inactivation; however, in the case of non-random or skewed X-inactivation, mild symptoms or clinical phenotypes resembling those seen in affected males are also observed in heterozygous female carriers [29]. To the best of our knowledge, Case 1 is the second female patient reported carrying an X-autosome translocation, with the der(X) breakpoint mapping upstream the *POU3F4* gene [30]. A highly skewed X-inactivation pattern was observed in Case 1 (Fig 1E); even though the X-inactivation analysis does not provide information on the identity of the inactivated chromosome, a favourable inactivation of the normal, non-translocated X chromosome is hypothesized. This was supported by the absence of a PCR product after amplification of the patient’s cDNA with *POU3F4* primer pairs (Fig 1D). Collectively, we suggest that the t(X;1) translocation in Case 1 disrupts the TAD structure of the *POU3F4* locus by physically dissociating the gene from important upstream otic enhancers regulating its expression (Fig 1C) [31]. Thus, absence of a functional *POU3F4* gene, caused by LRPE and skewed X-inactivation, underlies hearing loss seen in the ABT Case 1.

In the ABT Case 2 presenting with mild to moderate ID, WG-MPS detected cryptic rearrangement complexity including thirty-eight translocation breakpoints (Fig 2; S2 Table). Such complex chromosomal profiles resemble those seen in chromothripsis during which a single catastrophic event occurs followed by rearrangement of the broken segments in a random order and orientation [32]. In addition, depth of coverage analysis detected a heterozygous 12p12.1 deletion covering exons 1–3 of the *SOX5* gene (NM_006940.4) and flanking two translocation breakpoints (Fig 2A). This deletion was missed by previous array-CGH, highlighting the power of low-coverage WG-MPS in identifying smaller deletions below the resolution of detection of array-CGH. The *SOX5* gene encodes for the SRY-box 5 protein; which is involved in the generation of the cranial neural crest and the proper development of corticofugal neurons [33,34]. Heterozygous 12p12 deletions and a reciprocal t(11;12) translocation disrupting either *SOX5* alone or *SOX5* together with other genes have been reported in patients presenting with ID, global developmental delay, language and motor impairment, and mild dysmorphic facial features [35], which collectively characterize the neurodevelopmental disorder Lamb-Shaffer syndrome (OMIM-616803). *SOX5* is predicted as extremely intolerant to loss of function and copy number variants (pLI = 1.00) with a haploinsufficiency score of 0.74% [4]. The above together with a report by Lelieveld *et al*. (2016) identifying *SOX5* as a new ID gene from a meta-analysis of 2,104 whole-exome sequencing trios [36], strongly support that the ID phenotype in Case 2 is due to *SOX5* haploinsufficiency caused by the cryptic chromothripsis rearrangement.

In the ABT Case 3 with dysmorphic facial features, speech delay and a t(4;9)(q26;p24) translocation, WG-MPS successfully mapped the der(9) breakpoint directly within intron 10/10 of the *NFIB* gene (NM_001190737.1). *NFIB* encodes for Nuclear Factor I/B, a cellular transcription factor involved in lung maturation and brain development [37]. Additional studies demonstrated another important role of *NFIB* in the development of the pons, which is part of the brainstem, and pontine nuclei involved in the motor regulation of facial expressions, chewing, and swallowing [37,38]. In Latin, pons literally means bridge resembling its function serving as a connection between different parts of the brain, including the cerebral cortex and the cerebellum, both of which have important roles in speech development, as well as language- and other communication-related functions [39,40]. Since the pons is involved in the regulation of two main speech processes, i.e. respiration (mediated by the lungs) and articulation (mediated by movements of the jaw and mouth), it is therefore hypothesized that *NFIB* heterozygous disruption by the t(4;9) translocation causing dysfunction of the pons could underlie speech delay in Case 3. *NFIB* expression should be investigated next to determine the functional impact of the translocation; however, since its expression is very low in lymphocytes, an alternative tissue suitable to carry out this test cannot be obtained. Finally, a brain MRI would aid to visualize any hypodevelopment of the pons, thus further supporting a possible functional association between *NFIB* disruption and speech delay in Case 3.

In conclusion, WG-MPS utilized in this study proved to be a highly powerful universal method allowing rapid mapping of ABT breakpoints as well as identification of additional structural variants, independent of the chromosomes involved each time. Results from our recently published paper, demonstrated that translocations are coincidental and unrelated to phenotype presentation in the majority of familial ABT carriers with identical rearrangements and differential phenotypes [15]. In contrast, in line with previous reports and as demonstrated here, *de novo* translocations usually occur within or close to pathogenic genes and they seem to be associated with phenotype presentation in affected carriers via cryptic complexity, LRPE, and direct gene disruption. Collectively, this study highlights the efficacy of low coverage WG-MPS in accurately characterizing ABT breakpoints in both coding and non-coding regions, and suggesting underlying molecular mechanisms for phenotype association, thus supporting its potential introduction in the clinical investigation of ABTs.

## Supporting information

S1 Table List of PCR primers used for translocation breakpoint validation. The primer name, sequence, and melting temperature for each primer used for translocation breakpoint validation is given. The annealing temperature and extension time used as well as the approximate size of each amplicon, as compared with either a 100bp or 1kb DNA ladder, are also given.
(DOCX)

S2 Table Translocation breakpoint junctions of the chromothripsis rearrangement as identified by whole-genome mate-pair sequencing in Case 2. In the chromothripsis rearrangement, each translocation breakpoint split the chromosome into two fragments onto which fragments from the same or other chromosomes were joined onto. The table below includes the translocation breakpoint junctions on each chromosome as estimated by WG-MPS (1st column), the junction length (2nd column), as well as the translocation breakpoint junction fragments (3rd column) that joined on the left and right side of each fragment. In addition, the joining fragment pairs as illustrated in Fig 2B (4th column) as well as fragment orientations (5th column) are also given. Forward-Forward (FF) and Reverse-Reverse (RR) orientations indicate that one of the two joining fragments has been inverted.
(DOCX)

S1 Fig *POU3F4* mutation screening results in Case 1. A) Sanger sequencing electropherogram screenshot illustrating the two benign *POU3F4* SNPs identified in Case 1 (red arrows). B) Pairwise alignment of the nucleotide sequence including the two SNPs (bottom line) and the corresponding reference sequence (top line). The two mismatches indicating the SNP positions are indicated with dots.
(PDF)
