# Supplementary material for: Position effect, cryptic complexity, and direct gene disruption as disease mechanisms in de novo apparently balanced translocation cases
Source: PLoS One. 2018 Oct 5;13(10):e0205298. doi: 10.1371/journal.pone.0205298 (PMC6173455; doi:10.1371/journal.pone.0205298)
Supplement: S1 Table — The primer name, sequence, and melting temperature for each primer used for translocation breakpoint validation is given. The annealing temperature and extension time used as well as the approximate size of each amplicon, as compared with either a 100bp or 1kb DNA ladder, are also given. (DOCX) [file pone.0205298.s001.docx]

**Table S1. List of PCR primers used for translocation breakpoint validation.**

The primer name and sequence for each primer used for translocation breakpoint validation is given. The annealing temperature and extension time used as well as the approximate size of each amplicon, as compared with either a 100bp or 1kb DNA ladder, are also given.

| **Case** | **Primer name** | **Primer Sequence (5’→3’)** | **Annealing Temp. (^o^C)** | **Extension Time** | **Amplicon size** |
| --- | --- | --- | --- | --- | --- |
| Case 1 | der(X)F | AGGTGGGGATACAGATCCAA | 61 | 1min | ~900bp |
|  | der(X)R | AAAAGCCTGTGCTAGAAAGAAG | 61 | 1min |  |
|  | der(1)F | GAAGCCAGTTATTCCCAGTCC | 61 | 3min | ~3000bp |
|  | der(1)R | GAGCTTTATGGTTCCCCTTTTT | 61 | 3min |  |
| Case 2 | der(6)-1F | CCACCTCAGCTTGACACAAC | 61 | 2min | ~1050bp |
|  | der(6)-1R | CCAAAATTGTCATGGAACAAAA | 61 | 2min |  |
|  | der(6)-2F | CGCAATCAAGAAAGGACAGA | 61 | 2min | ~800bp |
|  | der(6)-2R | GCGGCAATGGGTGTACTTAT | 61 | 2min |  |
|  | der(6)-3F | TTCAGCTGTAAAATGCTTTGCT | 61 | 2min | ~1200bp |
|  | der(6)-3R | CAACAGGCTTCTTGGTGGA | 61 | 2min |  |
|  | der(7)-1F | GCATGCAAAGTTAGCCACAA | 61 | 2min | ~1100bp |
|  | der(7)-1R | TTGACAAAACGATGCAGAGG | 61 | 2min |  |
|  | der(7)-2F | ATCGGGATGCAGAGATACGA | 61 | 1min30s | ~300bp |
|  | der(7)-2R | GACCACAGGGTTTCTTGAGTG | 61 | 1min30s |  |
|  | der(7)-3F | GCAATGAAACATAACTCAATCAGG | 61 | 7min | ~4300bp |
|  | der(7)-3R | CACCGTAAGTGTGCTGTGAAA | 61 | 7min |  |
|  | der(8)-1F | CACCAAATAGCACACAATCCA | 61 | 2min | ~1100bp |
|  | der(8)-1R | CGTCTGCAATAAGTTGTTCAAAGT | 61 | 2min |  |
|  | der(8)-2F | GATTATCAAAGTAGTACATGGTCACTG | 61 | 2min | ~1500bp |
|  | der(8)-2R | CTCACATGCTGCCCAATAAA | 61 | 2min |  |
|  | der(8)-3F | TGCAAGTGCTTCAGGAAATG | 61 | 2min | ~1100bp |
|  | der(8)-3R | GTGGATTTTCTGCTCCTTGG | 61 | 2min |  |
|  | der(8)-4F | TGCACTCCATTTCCAATCAA | 61 | 2min | ~1500bp |
|  | der(8)-4R | GATGAGTAACCTCTCTCCAAAAGC | 61 | 2min |  |
|  | der(8)-5F | TCTGCACTCACGGTTTTCAG | 61 | 2min | ~900bp |
|  | der(8)-5R | TCCTCCACGCTATGGGAAT | 61 | 2min |  |
|  | der(12)-1F | CACAATTCCTTGCACATGGT | 61 | 1min30s | ~550bp |
|  | der(12)-1R | AACAGCATTTGACTCCCACA | 61 | 1min30s |  |
|  | der(12)-2F | CCCAGGTGCATGTTTACAGA | 61 | 2min | ~1500bp |
|  | der(12)-2R | AAGGAGCAACTCGTCAAACC | 61 | 2min |  |
|  | der(12)-3F | ATGATGGCTGGGTTTTTCTG | 61 | 1min30s | ~500bp |
|  | der(12)-3R | TCAAATTAGTATCCTTGATGGCTTT | 61 | 1min30s |  |
| Case 3 | der(4)F | CAGGGCATTTAATGAATTCTATGTT | 62 | 2min30s | ~2000bp |
|  | der(4)R | CCAAAATACTCACATGAAGGAAAA | 62 | 2min30s |  |
|  | der(9)F | GGACAAGAGACAAAGGCAAGT | 62 | 2min30s | ~2000bp |
|  | der(9)R | AAACAAAACCCTTCCATTTTCA | 62 | 2min30s |  |
