# Supplementary material for: Position effect, cryptic complexity, and direct gene disruption as disease mechanisms in de novo apparently balanced translocation cases
Source: PLoS One. 2018 Oct 5;13(10):e0205298. doi: 10.1371/journal.pone.0205298 (PMC6173455; doi:10.1371/journal.pone.0205298)
Supplement: S2 Table — In the chromothripsis rearrangement, each translocation breakpoint split the chromosome into two fragments onto which fragments from the same or other chromosomes were joined onto. The table below includes the translocation breakpoint junctions on each chromosome as estimated by WG-MPS (1st column), the junction length (2nd column), as well as the translocation breakpoint junction fragments (3rd column) that joined on the left and right side of each fragment. In addition, the joining fragment pairs as illustrated in Fig 2B (4th column) as well as fragment orientations (5th column) are also given. Forward-Forward (FF) and Reverse-Reverse (RR) orientations indicate that one of the two joining fragments has been inverted. (DOCX) [file pone.0205298.s002.docx]

**Table S2. Translocation breakpoint junctions of the chromothripsis rearrangement as identified by whole-genome mate-pair sequencing in Case 2.**

In the chromothripsis rearrangement, each translocation breakpoint split the chromosome into two fragments onto which fragments from the same or other chromosomes were joined onto. The table below includes the translocation breakpoint junctions on each chromosome as estimated by WG-MPS (1^st^ column), the junction length (2^nd^ column), as well as the translocation breakpoint junction fragments (3^rd^ column) that joined on the left and right side of each fragment. In addition, the joining fragment pairs as illustrated in Fig 2B (4^th^ column) as well as fragment orientations (5^th^ column) are also given. Forward-Forward (FF) and Reverse-Reverse (RR) orientations indicate that one of the two joining fragments has been inverted.

| **Translocation junction as estimated by WG-MPS** | **Junction Length** | **Translocation junction fragment joined on left (L) and right (R) side** | | **Fragments as in Fig 2B** | **Fragment Orientation** |
| --- | --- | --- | --- | --- | --- |
| chr6:16754266-16755111 | 845bp | L | chr8:132937706-132938936 | 6-1/8-7 | FF |
|  |  | R | chr7:125837049-125838526 | 6-2/7-14 | FR |
| chr7:117767865-117769118 | 1253bp | L | chr7:118190127-118191284 | 7-1/7-2 | FR |
|  |  | R | chr7:119972097-119973002 | 7-2/7-4 | RR |
| chr7:118190127-118191284 | 1157bp | L | chr7:117767865-117769118 | 7-2/7-1 | RF |
|  |  | R | chr7:119972097-119973002 | 7-3/7-5 | RF |
| chr7:119510701-119512025 | 1324bp | L | chr12:23078654-23079369 | 7-3/12-3 | RR |
|  |  | R | chr7:120530428-120531641 | 7-4/7-8 | RF |
| chr7:119972097-119973002 | 905bp | L | chr7:117767865-117769118 | 7-4/7-2 | RR |
|  |  | R | chr7:118190127-118191284 | 7-5/7-3 | FR |
| chr7:120348350-120349253 | 903bp | L | chr12:21548186-21555397 | 7-5/12-1 | FR |
|  |  | R | chr12:27469136-27470256 | 7-6/12-6 | RR |
| chr7:120415788-120417672 | 1884bp | L | chr7:122652087-122653915 | 7-6/7-9 | RR |
|  |  | R | chr8:138298956-138300081 | 7-7/8-9 | FR |
| chr7:120530428-120531641 | 1213bp | L | chr12:24016019-24017286 | 7-7/12-3 | FR |
|  |  | R | chr7:119510701-119512025 | 7-8/7-4 | FR |
| chr7:122652087-122653915 | 1828bp | L | chr7:125837049-125838526 | 7-8/7-13 | FR |
|  |  | R | chr7:120415788-120417672 | 7-9/7-6 | RR |
| chr7:124098564-124099825 | 1261bp | L | chr7:124545598-124548487 | 7-9/7-12 | RF |
|  |  | R | chr8:133213251-133214906 | 7-10/8-8 | FR |
| chr7:124121849-124122971 | 1122bp | L | chr8:132899241-132900431 | 7-10/8-6 | FF |
|  |  | R | chr12:27177142-27177824 | 7-11/12-6 | FR |
| chr7:124245517-124246377 | 860bp | L | chr7:131296372-131297913 | 7-11/7-16 | FF |
|  |  | R | chr8:132937706-132938936 | 7-12/8-6 | FF |
| chr7:124545598-124548487 | 2889bp | L | chr7:124098564-124099825 | 7-12/7-9 | FR |
|  |  | R | chr12:55357036-55357802 | 7-13/12-8 | RR |
| chr7:125837049-125838526 | 1477bp | L | ch7:122652087-122653915 | 7-13/7-8 | RF |
|  |  | R | chr6:16754266-16755111 | 7-14/6-2 | RF |
| chr7:126465709-126468406 | 2697bp | L | chr7:131296372-131297913 | 7-14/7-15 | RF |
|  |  | R | chr8:131433335-131434497 | 7-15/8-2 | FF |
| chr7:131296372-131297913 | 1541bp | L | chr7:126465709-126468406 | 7-15/7-14 | FR |
|  |  | R | chr7:124245517-124246377 | 7-16/7-11 | FF |
| chr8:129588349-129589617 | 1268bp | L | chr12:55357036-55357802 | 8-1/12-9 | FF |
|  |  | R | chr12:24124507-24124723 | 8-2/12-5 | FR |
| chr8:131433335-131434497 | 1162bp | L | chr7:126465709-126468406 | 8-2/7-15 | FF |
| chr8:132197337-132200470 | 133bp | R | chr12:64836498-64837586 | 8-4/12-10 | RR |
| chr8:132602313-132603246 | 933bp | L | chr12:65386366-65387285 | 8-4/12-13 | RR |
|  |  | R | chr12:68784019-68784572 | 8-5/12-14 | FR |
| chr8:132899241-132900431 | 1190bp | L | chr12:27177142-27177824 | 8-5/12-5 | FR |
|  |  | R | chr7:124121849-124122971 | 8-6/7-10 | FF |
| chr8:132937706-132938936 | 1230bp | L | chr7:124245517-124246377 | 8-6/7-12 | FF |
|  |  | R | chr6:16754266-16755111 | 8-7/6-1 | FF |
| chr8:133213251-133214906 | 1655bp | L | chr12:27469136-27470256 | 8-7/12-7 | FF |
|  |  | R | chr7:124098564-124099825 | 8-8/7-10 | RF |
| chr8:138298956-138300081 | 1125bp | L | chr12:64792029-64792727 | 8-8/12-9 | RF |
|  |  | R | chr7:120415788-120417672 | 8-9/7-7 | RF |
| chr8:138551547-138552330 | 783bp | L | chr12:65246963-65248542 | 8-9/12-12 | RR |
|  |  | R | chr12:23078654-23079369 | 8-10/12-2 | FF |
| chr12:21548186-21555397 | 7211bp | L | chr7:120348350-120349253 | 12-1/7-5 | RF |
|  |  | R | chr12:55247926-55249292 | 12-2/12-7 | FF |
| chr12:23078654-23079369 | 715bp | L | chr8:138551547-138552330 | 12-2/8-10 | FF |
|  |  | R | chr7:119510701-119512025 | 12-3/7-3 | RR |
| chr12:24016019-24017286 | 1267bp | L | chr7:120530428-120531641 | 12-3/7-7 | RF |
| chr12:24124507-24124723 | 216bp | R | chr8: 129588349-129589617 | 12-5/8-2 | RF |
| chr12:27177142-27177824 | 682bp | L | chr8: 132899241-132900431 | 12-5/8-5 | RF |
|  |  | R | chr7: 124121849-124122971 | 12-6/7-11 | RF |
| chr12:27469136-27470256 | 1120bp | L | chr7: 120348350-120349253 | 12-6/7-6 | RR |
|  |  | R | chr8: 133213251-133214906 | 12-7/8-7 | FF |
| chr12:55247926-55249292 | 1366bp | L | chr12: 21548186-21555397 | 12-7/12-2 | FF |
|  |  | R | chr12: 68784019-68784572 | 12-8/12-13 | RR |
| chr12:55357036-55357802 | 766bp | L | chr7: 124545598-124548487 | 12-8/7-13 | RR |
|  |  | R | chr8: 129588349-129589617 | 12-9/8-1 | FF |
| chr12:64792029-64792727 | 698bp | L | chr8: 138298956-138300081 | 12-9/8-8 | FR |
|  |  | R | chr12: 64836498-64837586 | 12-10/12-11 | RF |
| chr12:64836498-64837586 | 1088bp | L | chr8: 132197337-132200470 | 12-10/8-4 | RR |
|  |  | R | chr12: 64792029-64792727 | 12-11/12-10 | FR |
| chr12:65246963-65248542 | 1579bp | L | chr12: 65386366-65387285 | 12-11/12-12 | FR |
|  |  | R | chr8: 138551547-138552330 | 12-12/8-9 | RR |
| chr12:65386366-65387285 | 919bp | L | chr12: 65246963-65248542 | 12-12/12-11 | RF |
|  |  | R | chr8: 132602313-132603246 | 12-13/8-4 | RR |
| chr12:68784019-68784572 | 553bp | L | chr12: 55247926-55249292 | 12-13/12-8 | RR |
|  |  | R | chr8: 132602313-132603246 | 12-14/8-5 | RF |
