# Supplementary material for: Position effect, cryptic complexity, and direct gene disruption as disease mechanisms in de novo apparently balanced translocation cases
Source: PLoS One. 2018 Oct 5;13(10):e0205298. doi: 10.1371/journal.pone.0205298 (PMC6173455; doi:10.1371/journal.pone.0205298)
Supplement: S1 Fig — A) Sanger sequencing electropherogram screenshot illustrating the two benign POU3F4 SNPs identified in Case 1 (red arrows). B) Pairwise alignment of the nucleotide sequence including the two SNPs (bottom line) and the corresponding reference sequence (top line). The two mismatches indicating the SNP positions are indicated with dots. (PDF) [file pone.0205298.s003.pdf]

A)

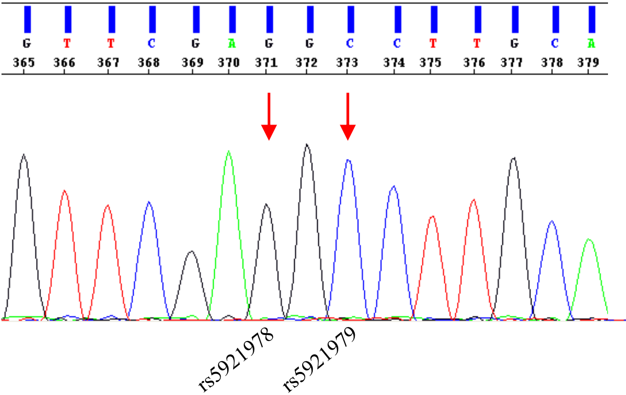

B)

```
  1 CAGGCCGACGTGGGGTTGGCGCTGGGCACACTGTATGGTAACGTGTTCTC  50
    ||||||||||||||||||||||||||||||||||||||||||||||||||
  1 CAGGCCGACGTGGGGTTGGCGCTGGGCACACTGTATGGTAACGTGTTCTC  50

 51 GCAGACCACCATCTGCAGGTTCGAAGGCTTGCAGCTGAGCTTCAAAAATA 100
    |||||||||||||||||||||||||.|.||||||||||||||||||||||
 51 GCAGACCACCATCTGCAGGTTCGAGGCCTTGCAGCTGAGCTTCAAAAATA 100

101 TGTGCAAGCTGAAGCCCCTGCTGAACAAGTGGCTGGAGGAGGCGGATTCG 150
    ||||||||||||||||||||||||||||||||||||||||||||||||||
101 TGTGCAAGCTGAAGCCCCTGCTGAACAAGTGGCTGGAGGAGGCGGATTCG 150
```
